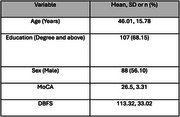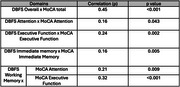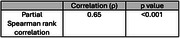# Construct Validity and Test‐Retest Reliability of the Digital Brain Function Screen (DBFS) for Early Cognitive Impairment

**DOI:** 10.1002/alz70857_098958

**Published:** 2025-12-24

**Authors:** See Ann Soo, Jermyn Z See, Nav Vij, Prem Pillay

**Affiliations:** ^1^ Neurowyzr Pte Ltd, Singapore, Singapore, Singapore; ^2^ Singapore Brain and Spine Specialist Singapore Brain‐Spine‐Nerves Center, Singapore, Singapore, Singapore

## Abstract

**Background:**

The Digital Brain Function Screen (DBFS) is a medical‐grade digital cognitive screening tool designed to detect early stages of Mild Cognitive Impairment (MCI) and monitor cognitive decline. While DBFS has shown comparability to the Montreal Cognitive Assessment (MoCA), a gold‐standard tool for MCI detection, its construct validity and test‐retest reliability had not been formally established.

The aim of this study was to evaluate the construct validity and test‐retest reliability of DBFS in comparison to MoCA.

**Method:**

A total of 157 individuals aged 10 to 81 years were recruited from a neurology clinic, with 36 participants completing a second DBFS assessment. Both DBFS and MoCA were administered during the same session, with an average interval between the first and second DBFS administrations of 305.33 ± 354.19 days (range: 1–1090 days). Construct validity was assessed using partial Spearman's rank correlations between domain‐specific DBFS and MoCA scores, controlling for covariates including age, sex, and education. Test‐retest reliability was evaluated by comparing DBFS scores from the first and second administrations using partial Spearman's rank correlation, while controlling for the same covariates. Statistical analysis was performed using R (https://www.r‐project.org/).

**Result:**

The DBFS total score was significantly correlated with the MoCA total score (*p* < 0.001), and strong positive correlations (*p* < 0.05) were observed between DBFS domains and corresponding MoCA domains, indicating good construct validity. Among the 36 participants who completed the DBFS a second time, Spearman's ρ was 0.65 (*p* < 0.001), demonstrating a strong monotonic relationship and high reliability over time.

**Conclusion:**

The study confirms that DBFS domains effectively measure the intended cognitive constructs and maintain high reliability across repeated administrations. These findings establish the validity and reliability of DBFS as a practical, scalable, and efficient tool for detecting and monitoring early cognitive impairment. Its usability makes it suitable for deployment in various healthcare settings, including screening centers and specialty clinics.